# Endocan Participates in TGF-β/Smad and AKT/ERK Signaling Coordination and lncRNA Modulation in Human Cardiac Fibroblasts

**DOI:** 10.3390/ijms27177745

**Published:** 2026-08-29

**Authors:** Federica Aliquò, Alice Pantano, Giulia Giuffrè, Davide Labellarte, Angela Avenoso, Adele Campo, Giovanna Vermiglio, Giuseppe M. Campo, Angela D’Ascola, Michele Scuruchi

**Affiliations:** 1 Department of Biomedical and Dental Sciences and Morphofunctional Images, University of Messina, Via Consolare Valeria 1, 98125 Messina, Italy; faliquo@unime.it (F.A.); aavenoso@unime.it (A.A.); giovanna.vermiglio1@unime.it (G.V.); 2 Department of Clinical and Experimental Medicine, University of Messina, Via Consolare Valeria 1, 98125 Messina, Italy; alice.pantano@unime.it (A.P.); adycampo96@gmail.com (A.C.); giuseppe.campo@unime.it (G.M.C.); 3 Department of Mathematical and Computer Sciences, Physical Sciences and Earth Sciences, University of Messina, Viale Ferdinando Stagno D’Alcontres 31, 98166 Messina, Italy; giulia.giuffre00@gmail.com; 4 Department of Chemical, Biological, Pharmaceutical and Environmental Sciences, University of Messina, Viale Ferdinando Stagno D’Alcontres 31, 98166 Messina, Italy; davide.labellarte@unime.it

**Keywords:** cardiac fibrosis, endocan (ESM-1), proteoglycans, TGF-β signaling coordination, lncRNAs, myofibroblast transdifferentiation

## Abstract

Endocan, a soluble dermatan sulfate proteoglycan, is increasingly recognized as a regulator of key cellular pathways in both physiological and pathological contexts. While TGF-β is a central mediator of fibrotic remodeling, the role of endocan in this process remains elusive. By performing a transcriptomic dataset analysis [GSE116250], we found that endocan expression is upregulated and correlates with fibrotic markers in human failing hearts. We investigated the role of endocan in an in vitro model of cardiac fibroblasts stimulated with TGF-β. Our results identified endocan as a TGF-β-responsive gene in primary human cardiac fibroblasts. Indeed, by inhibiting endocan expression using a specific siRNA, we demonstrated that this proteoglycan is required for the full expression of key fibrosis-related genes (Col1a1, α-SMA, MMP-3, and MMP-9) and modulates the expression of long non-coding RNAs (MALAT1, H19 and HOTAIR), which are essential for the epigenetic control of the fibroblast phenotype. These effects depend on the modulation of both canonical (SMAD3) and non-canonical (AKT and ERK1/2) TGF-β pathways. Taken together, these findings indicate that endocan is required for the coordinated activation of canonical and non-canonical TGF-β signaling during fibroblast transdifferentiation and suggest its potential involvement in the molecular mechanisms underlying cardiac fibrosis.

## 1. Introduction

Transforming growth factor (TGF)-β is a multifunctional cytokine expressed by almost every tissue and cell type. It regulates a wide range of cellular processes, including cell survival, metabolism, growth, proliferation, differentiation, adhesion, migration, and cell death in different cell types and in a context-dependent manner [1]. Embryonic development, wound healing, tissue homeostasis, and immune regulation are also regulated by this cytokine [1]. However, dysregulation of TGF-β signaling is implicated in multiple pathological conditions, including immune dysfunction, cancer, and fibrosis. The complexity of TGF-β’s action arises from its capacity to orchestrate intracellular signaling through both canonical Smad-dependent and non-canonical, Smad-independent signaling pathways that contribute to diverse cellular responses in several tissues and contexts [1].

A well-established consequence of TGF-β dysregulation is the promotion of epithelial–mesenchymal transition (EMT) and excessive extracellular matrix (ECM) deposition, which contribute to the progression of fibrosis [2,3].

Among the ECM-regulatory enzymes induced during fibrotic remodeling, matrix metalloproteinases (MMPs) play a central role by degrading structural ECM components and by releasing bioactive molecules, including latent TGF-β itself, thereby amplifying the fibrotic response [4]. Among the MMP family, MMP-3 and MMP-9 were selected in this study as representative members with well-documented TGF-β responsiveness during cardiac remodeling, although other MMPs, particularly MMP-2, are also extensively studied in the cardiovascular field and may play complementary roles in TGF-β-driven fibrotic processes. In parallel with ECM-degrading activity, TGF-β also directly promotes the transcription of fibrillar collagen genes: upon activation, Smad3 forms a complex with Smad4 that translocates to the nucleus and binds directly to Smad-binding elements within collagen gene promoters, driving their transcription, an effect specifically demonstrated in cardiac fibroblasts [5]. Together, these degradative and biosynthetic processes are tightly coordinated to sustain the progressive accumulation of fibrotic ECM.

Endocan, also known as endothelial-specific molecule-1 (ESM-1), is a small dermatan sulfate (DS) proteoglycan (PG), originally identified as a circulating PG constitutively secreted by endothelial cells [6]. However, it is also found in other cell types, including human adipocytes [7], melanoma cells [8], and chondrocytes [9]. Typically, endocan expression is upregulated in highly proliferative tissues and cells and is limited in quiescent cells, positioning it as a key regulator in multiple pathological conditions with tissue remodeling [10], consistent with the broader recognition of proteoglycans as multifunctional regulators of cell signaling and matrix homeostasis [11,12]. Notably, endocan has emerged as a key player in cardiovascular disease, with growing evidence supporting its role as both a biomarker and a functional mediator of endothelial dysfunction and vascular remodeling [13].

Importantly, endocan gene expression is regulated by growth factors, including VEGF-A, VEGF-C, EGFR, and TGF-β, as well as proinflammatory cytokines, including TNF-α and IL-1β [14,15]. These factors activate complex signaling pathways like NF-kB, PI3K/Akt, PKC, and ERK1/2, which enhance endocan expression and contribute to its role in regulating inflammation, cell proliferation, angiogenesis, and tumor progression [9,16,17,18,19].

Long non-coding RNAs (lncRNAs) are a class of functional molecules with lengths of more than 200 nt that finely regulate important biological functions, such as the cell cycle and apoptosis, inflammatory responses, cellular metabolism, and tissue homeostasis [20,21]. Due to these abilities, many lncRNAs, including HOTAIR, H19, and MALAT1, are recognized as epigenetic regulators with a crucial role in the development of various diseases, including fibrosis [22,23,24,25,26].

Interestingly, it has been demonstrated that elevated serum endocan levels are present in nonalcoholic fatty liver disease (NAFLD) and advanced liver fibrosis [27,28]. Furthermore, both in vivo and in vitro models of renal fibrosis, endocan overexpression has been associated with increased expression of various mesenchymal and fibrotic markers, indicating that endocan may promote EMT in renal fibrosis [29].

Given the known effects of TGF-β in promoting fibrotic responses and the observed levels of endocan in liver and renal fibrosis, it is plausible that TGF-β signaling pathways may influence endocan expression during the fibrotic process. To address this hypothesis, we first analyzed a publicly available transcriptomic dataset of failing human hearts to assess endocan expression and its correlation with established fibrotic markers. Building on this, we investigated endocan mRNA expression and related protein levels in cardiac fibroblasts stimulated with TGF-β. Furthermore, we performed endocan knockdown to study the effects of increased endocan expression on TGF-β signaling. Additionally, we also studied the effects of endocan knockdown on H19, MALAT1, and HOTAIR, three lncRNAs regulated by TGF-β.

## 2. Results

### 2.1. Endocan Expression Is Upregulated and Correlates with Fibrotic Markers in Human Failing Hearts

**Figure 1:**
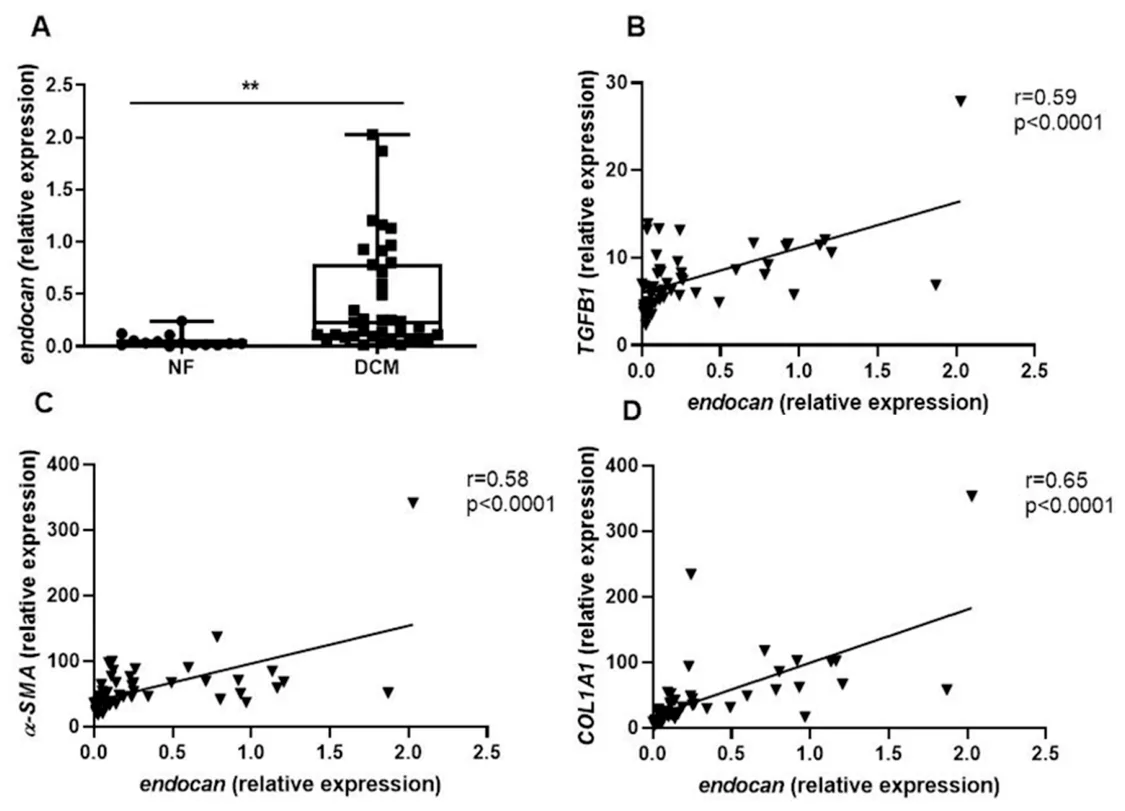
Endocan expression is upregulated in human failing hearts and correlates with fibrotic markers. (**A**) Relative mRNA expression of endocan in left ventricle (LV) samples from non-failing donors (NF, *n* = 14) and patients with Dilated Cardiomyopathy (DCM, n = 37), based on the secondary analysis of the GSE116250 dataset. Individual data points are shown, and the box plot represents the median and interquartile range. Statistical significance was determined by Student’s *t*-test. ** *p* < 0.01 vs. NF. (**B**–**D**) Pearson’s correlation analysis between endocan mRNA levels and key fibrotic markers: (**B**) TGFB1, (**C**) α-SMA, and (**D**) COL1A1. The Pearson correlation coefficient (r) and *p*-value are indicated in each panel.

To investigate the clinical relevance of endocan in cardiac fibrosis, we first performed a secondary analysis of the GSE116250 transcriptomic dataset. Our results showed that endocan mRNA levels were significantly upregulated in the left ventricles of patients with Dilated Cardiomyopathy (DCM) compared to non-failing (NF) donors (Figure 1A). Furthermore, we found a strong and significant positive correlation between endocan expression and key markers of cardiac fibrosis, including TGFB1 (r = 0.59, *p* < 0.0001), ACTA2 (α-SMA) (r = 0.58, *p* < 0.0001), and COL1A1 (r = 0.65, *p* < 0.0001) (Figure 1B–D). These correlations remained virtually unchanged after adjustment for age and sex (TGFB1: r = 0.597; ACTA2: r = 0.583; COL1A1: r = 0.653; all *p* < 0.0001) and were robust to the exclusion of influential observations identified by sensitivity analysis (all *p* < 0.005), confirming that the associations are not attributable to clinical confounders or to a small number of extreme values (Supplementary Table S1). These findings suggest that endocan is a relevant player in the fibrotic remodeling of the human heart, pointing to its potential role as a mediator of fibroblast activation.

### 2.2. TGF-β Induces Endocan Expression and Secretion in Human Cardiac Fibroblasts

**Figure 2:**
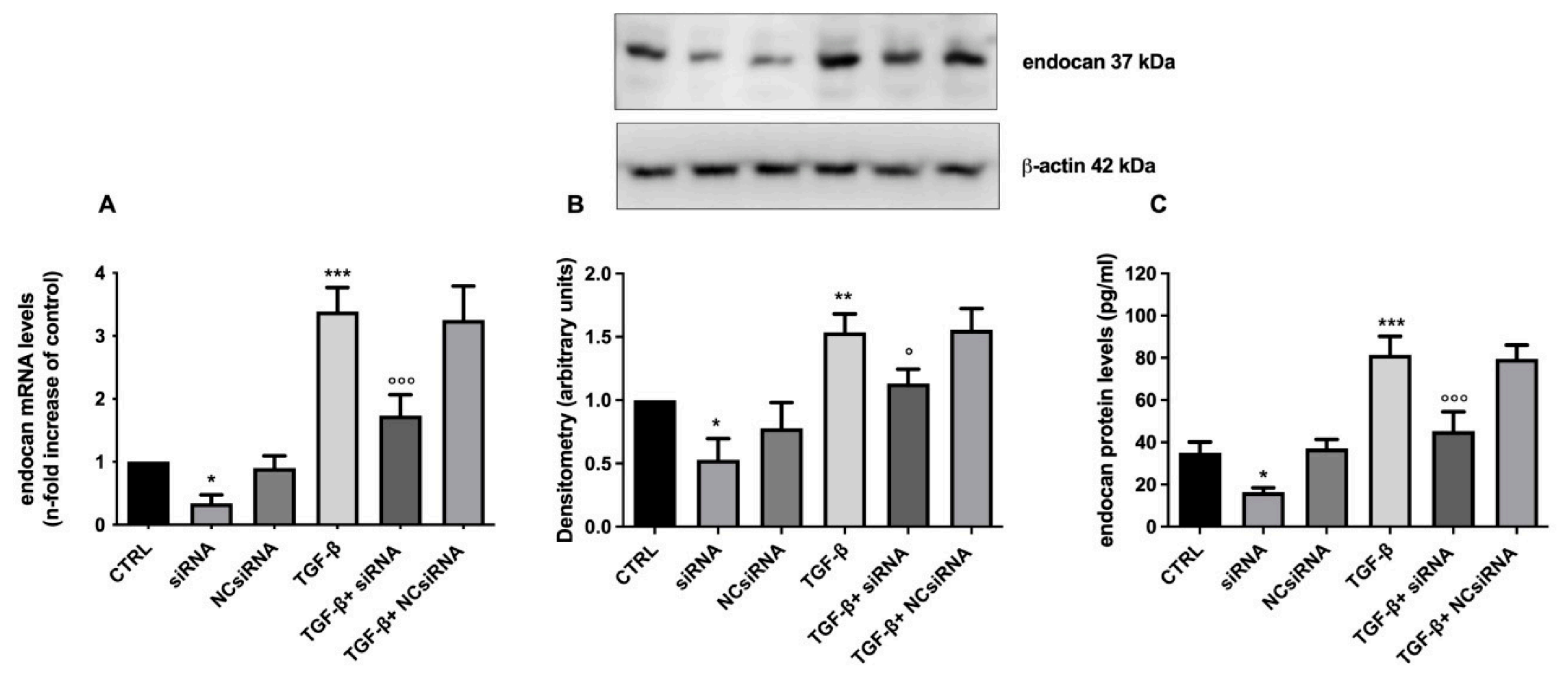
TGF--β induces endocan expression and secretion in human cardiac fibroblasts. Human cardiac fibroblasts were left untreated (CTRL), stimulated with TGF-β (10 ng/mL, 24 h), transfected with a non-targeting negative control siRNA alone (NC-siRNA) or in combination with TGF-β (NC-siRNA + TGF-β), or transfected with an endocan-specific siRNA prior to TGF-β stimulation (endocan-siRNA + TGF-β). Endocan mRNA levels (**A**) were quantified by qPCR and are expressed as fold change relative to CTRL. Intracellular protein levels (**B**) were measured by Western blotting and expressed as arbitrary units. Secreted endocan (**C**) was measured by ELISA in the culture media and expressed as pg/mL. Data are displayed as mean ± SD of at least five (**A**,**C**) or three (**B**) independent experiments. *** *p* < 0.001, ** *p* < 0.01 and * *p* < 0.05 vs. CTRL, °°° *p* < 0.001 and ° *p* < 0.05, vs. TGF-β.

To validate the role of endocan in a functional model, human cardiac fibroblasts were assigned to the following experimental groups: untreated cells (CTRL), cells stimulated with TGF-β (10 ng/mL) alone for 24 h, cells transfected with a non-targeting negative control siRNA (NC-siRNA) alone, cells transfected with NC-siRNA and subsequently stimulated with TGF-β (NC-siRNA + TGF-β), and cells transfected with an endocan-specific siRNA prior to TGF-β stimulation (endocan-siRNA + TGF-β). As shown in Figure 2, TGF-β exposure induced an approximately 3.5-fold increase in endocan mRNA compared with CTRL (panel A). Consistently, Western blot analysis revealed a significant increase in intracellular endocan protein levels in TGF-β-stimulated cells relative to CTRL (panel B). Moreover, ELISA analysis showed a marked increase in secreted endocan levels in the culture medium of TGF-β-treated cells (approximately 85 pg/mL) compared with CTRL (approximately 35 pg/mL) (panel C), indicating that this PG is actively produced and released upon TGF-β stimulation in cardiac fibroblasts. Conversely, endocan knockdown reversed this trend, significantly reducing both intracellular and extracellular endocan levels even in the presence of TGF-β stimulation. Endocan-specific siRNA transfection alone achieved a silencing efficiency of approximately 62% in endocan mRNA levels compared with NC-siRNA-treated cells (and approximately 66% compared with untreated CTRLs), confirming the effectiveness of the knockdown approach. Importantly, NC-siRNA, alone or in combination with TGF-β, did not exert any effect compared with the respective CTRL and TGF-β-alone groups, confirming the specificity of the silencing approach (Supplementary Table S2 and Figure S1).

### 2.3. Endocan Promotes Myofibroblast Activation by Modulating the Canonical TGF-β/SMAD3 Signaling Pathway

**Figure 3:**
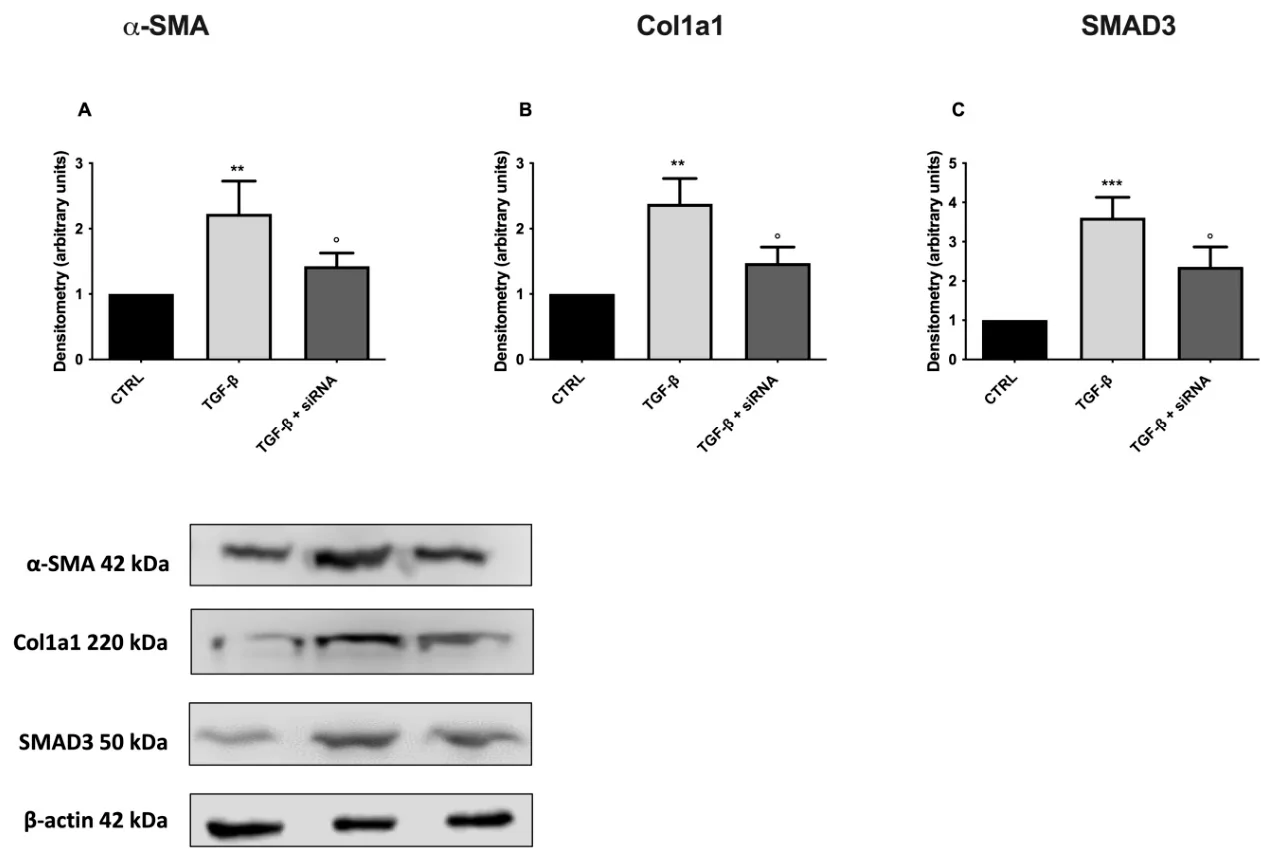
Endocan knockdown attenuates myofibroblast activation and inhibits TGF-β signaling pathways. Protein levels of α-SMA (**A**), COL1A1 (**B**), and p-SMAD3 (**C**) were measured by Western blotting analysis in cardiac fibroblasts. Data are displayed as the mean ± SD of three experiments and are expressed as arbitrary units. *** *p* < 0.001 and ** *p* < 0.01 vs. CTRL, ° *p* < 0.05 vs. TGF-β.

To investigate the molecular mechanisms by which endocan influences fibroblast activation, we analyzed both canonical (SMAD-dependent) and non-canonical (SMAD-independent) TGF-β signaling pathways. As shown in Figure 3, TGF-β stimulation induced a robust increase in the protein levels of α-SMA and Col1a1, well-known markers of myofibroblast differentiation (Panels A and B). Notably, endocan knockdown significantly blunted the expression of these fibrotic markers, suggesting a pivotal role for this PG in myofibroblast activation. This phenotypic reversal was associated with a significant modulation of intracellular signaling; indeed, Western blot analysis revealed that endocan silencing markedly reduced TGF-β-induced phosphorylation of SMAD3 (Panel C).

**Figure 4:**
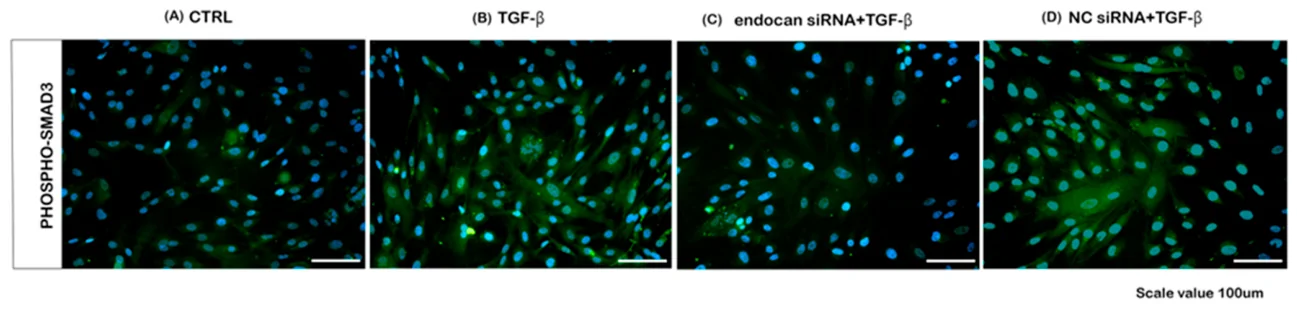
Compound panel of immunofluorescence reactions using an anti- p-SMAD3 monoclonal antibody (green fluorescence). Control cells exhibited a weak fluorescence staining pattern around the nuclei (**A**). In fibroblasts treated with TGF-β, the signal was increased in the perinuclear zone and at the cellular extension level (**B**). In fibroblasts where endocan knockdown was induced, the p-SMAD3 fluorescence was strongly reduced, even following TGF-β stimulation (**C**). Cells transfected with NC-siRNA and stimulated with TGF-β showed a staining pattern comparable to that of TGF-β-treated cells (**D**), confirming the specificity of the silencing effect. Images were acquired at a magnification of 20×; scale bar = 100 μm.

These findings were further confirmed by investigating p-SMAD3 via immunofluorescence (Figure 4). As reported, the fluorescence pattern of p-SMAD3 was visibly increased in TGF-β-stimulated fibroblasts (panel B). Notably, cells transfected with NC-siRNA and stimulated with TGF-β (Panel D) exhibited a fluorescence pattern comparable to cells treated with TGF-β alone, confirming that the transfection process itself did not interfere with the signaling pathway. On the contrary, in endocan knockdown cells, this signal appeared markedly reduced (panel C). These data suggest that endocan is specifically required for the activation of TGF-β/SMAD3 signaling, thereby affecting fibroblast activation and transdifferentiation (Supplementary Table S2 and Figure S1).

### 2.4. Endocan Knockdown Modulates the Non-Canonical TGF-β Pathways, Reducing AKT and ERK1/2 Activation

**Figure 5:**
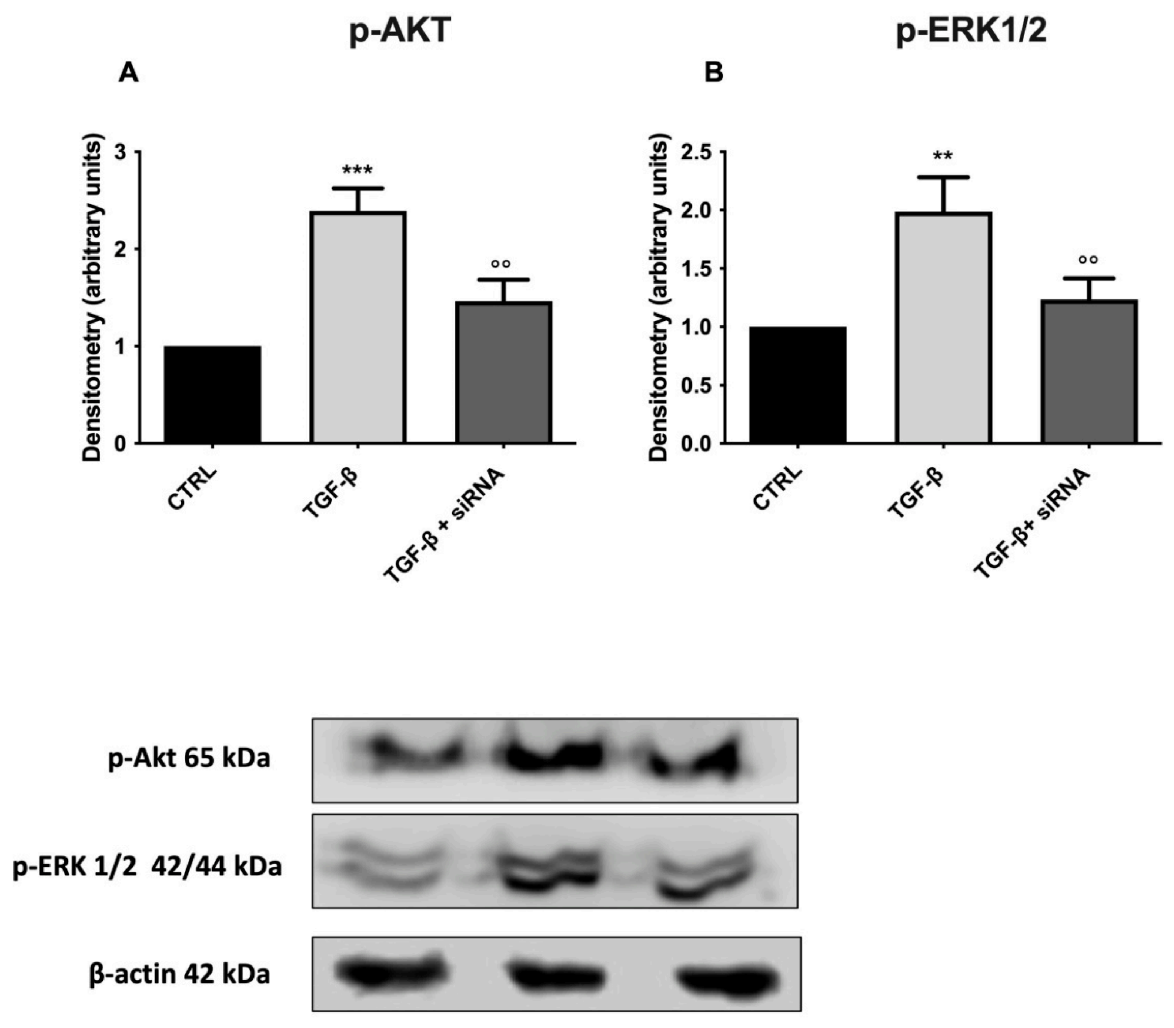
Endocan knockdown inhibits non-canonical TGF-β signaling pathways. (**A**) p-AKT protein levels in cardiac fibroblasts; (**B**) p-ERK 1/2 protein levels in cardiac fibroblasts. Both are expressed as fold-change relative to untreated control (CTRL) cells, representing basal signaling activity, and were measured by Western blotting analysis. Data are displayed as the mean ± SD of three experiments and are expressed as arbitrary units. *** *p* < 0.001 and ** *p* < 0.01 vs. CTRL; °° *p* < 0.01 vs. TGF-β.

TGF-β promotes its signaling through the activation of the TGF-β/Smad classical signaling pathway and by regulating non-classical signaling pathways, including the PI3K/AKT and the ERK1/2 pathways. Figure 5 reports the results obtained by evaluating the p-AKT and p-ERK 1/2 levels in cardiac fibroblasts. As shown, p-AKT levels were significantly increased when cells were stimulated with TGF-β; interestingly, in endocan knockdown cells activated by TGF-β, p-AKT levels were significantly decreased compared with cells treated with TGF-β alone (panel A). Similar effects were observed when evaluating p-ERK 1/2 (panel B).

**Figure 6:**
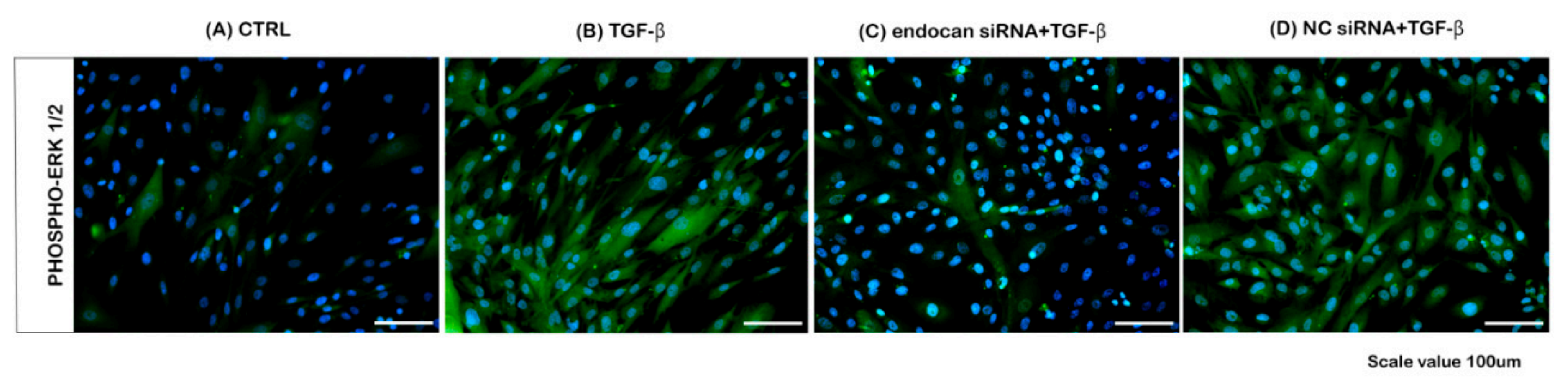
Compound panel of immunofluorescence reactions using an anti- p-ERK1/2 polyclonal antibody (green fluorescence). The control cells exhibited a weak fluorescence staining pattern around the nuclei (**A**). In fibroblasts treated with TGF-β, the signal was increased in the perinuclear zone and at the cellular extension level (**B**). In fibroblasts where endocan knockdown was induced, the p-ERK1/2 fluorescence was strongly reduced (**C**). Cells transfected with NC-siRNA and stimulated with TGF-β (**D**) showed a staining pattern comparable to that of TGF-β-treated cells, confirming the specificity of the silencing effect. Images were acquired at a magnification of 20×. Scale bar = 100 μm.

These findings were further confirmed by investigating p-ERK1/2 via immunofluorescence (Figure 6). As reported, the p-ERK1/2 fluorescence pattern was visibly increased in TGF-β-stimulated fibroblasts (panel B). Notably, in cells transfected with NC-siRNA and stimulated with TGF-β (panel D), the fluorescence signal remained comparable to that in cells treated with TGF-β only, confirming the specificity of the treatment. In contrast, in endocan knockdown cells, this signal appeared markedly reduced (panel C; Supplementary Table S2 and Figure S1).

### 2.5. Endocan Knockdown Attenuates TGF-β-Induced ECM Remodeling by Downregulating MMP-3 and MMP-9

**Figure 7:**
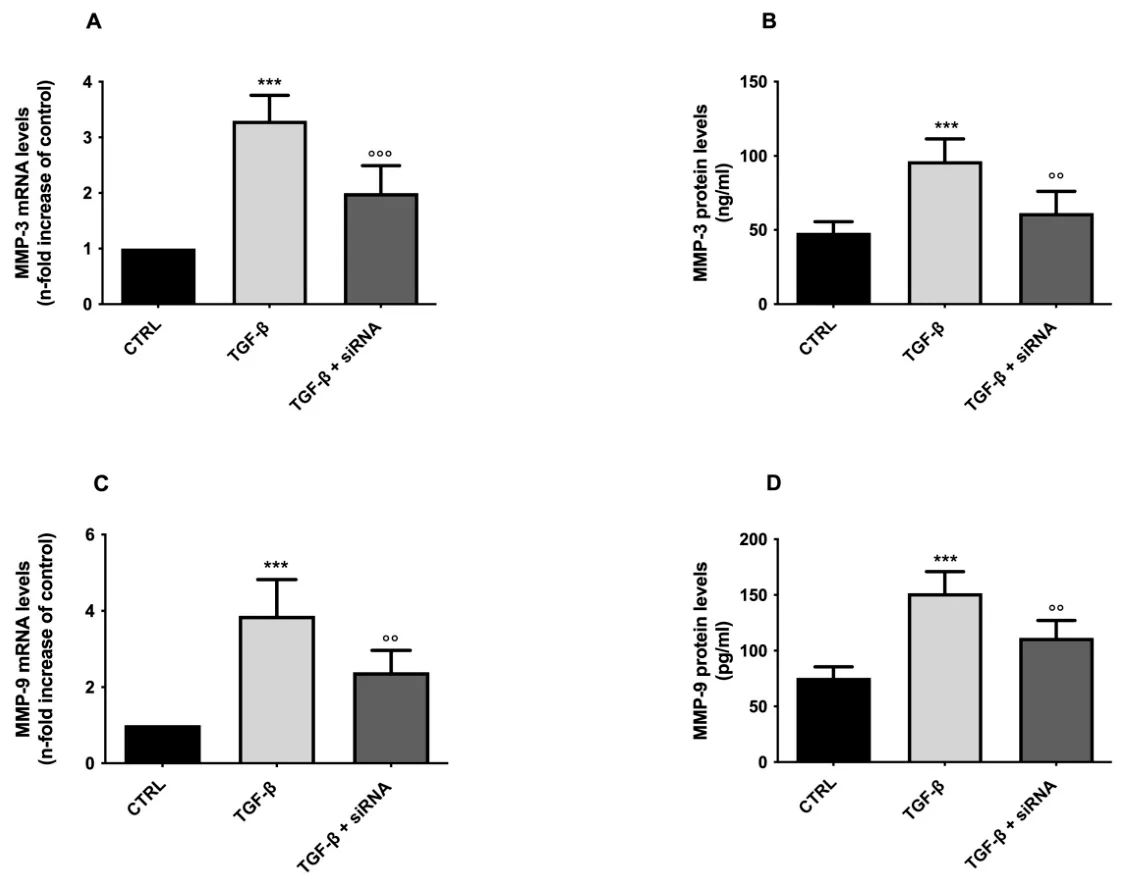
Endocan knockdown prevents TGF-β-induced upregulation of MMP-3 and MMP-9. mRNA levels of MMP-3 (**A**) and MMP-9 (**C**) were quantified by qPCR and are expressed as relative fold change. Protein levels of MMP-3 (**B**) and MMP-9 (**D**) were measured by ELISA in the culture media and expressed as ng/mL and pg/mL, respectively. Data are displayed as the mean ± SD of five independent experiments. *** *p* < 0.001 vs. CTRL, °°° *p* < 0.001, and °° *p* < 0.01 vs. TGF-β.

To evaluate the functional impact of endocan on ECM homeostasis, we analyzed the expression of MMPs, which play a crucial role in myocardial remodeling. As shown in Figure 7, TGF-β stimulation led to a robust upregulation of both MMP-3 and MMP-9 at the mRNA (panels A and C) and protein levels (panels B and D), respectively.

Interestingly, in cardiac fibroblasts transfected with endocan siRNA, the TGF-β-induced increase in MMP-3 and MMP-9 was markedly attenuated at both transcriptional and translational levels compared with cells treated with TGF-β alone. Since these MMPs are key drivers of collagen turnover and fibrotic progression, these findings indicate that endocan is an essential mediator of TGF-β-driven ECM remodeling in human cardiac fibroblasts (Supplementary Table S2).

### 2.6. Endocan Silencing Reverses the TGF-β-Induced Epigenetic Remodeling Mediated by lncRNAs

**Figure 8:**
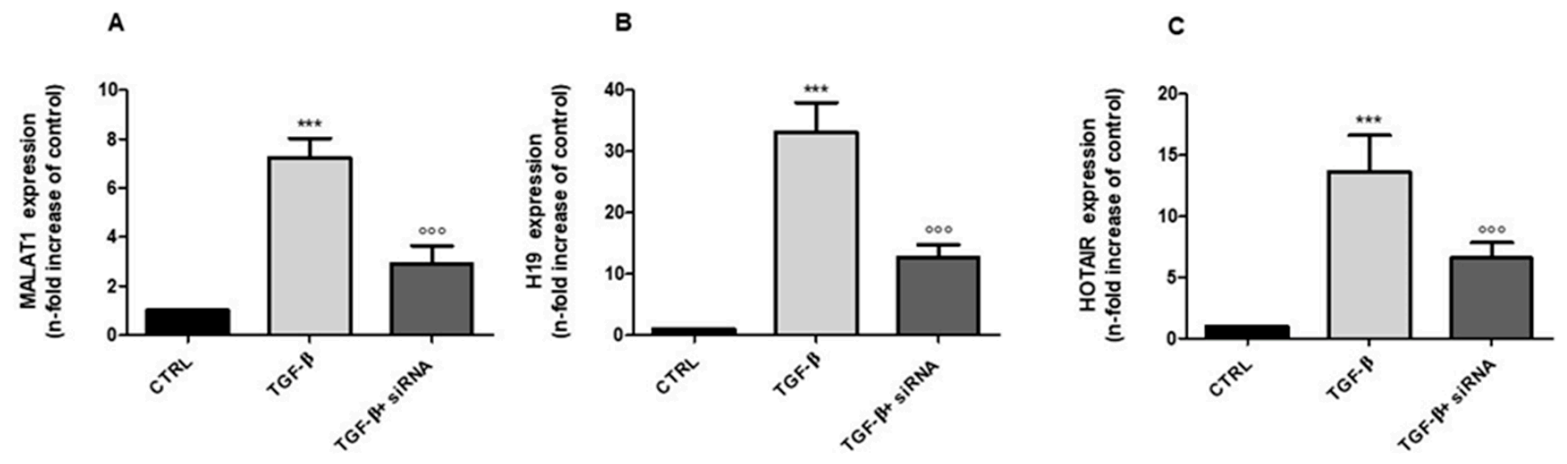
Endocan is required for the TGF-β-dependent induction of pro-fibrotic lncRNAs. Effects of TGF-β and endocan siRNA treatment on MALAT1 (**A**), H19 (**B**), and HOTAIR (**C**) expression in cardiac fibroblasts. Transcript levels were quantified by qPCR and are expressed as relative fold-change. Data are displayed as the mean ± SD of five independent experiments. *** *p* < 0.001 vs. CTRL; °°° *p* < 0.001 vs. TGF-β.

To further explore the regulatory depth of endocan in cardiac fibrosis, we investigated its influence on long non-coding RNAs (lncRNAs) known to orchestrate myofibroblast activation. As shown in Figure 8, TGF-β stimulation triggered a considerable upregulation of MALAT1 (~7.2-fold), H19 (~33-fold), and HOTAIR (~13.5-fold) (panels A, B, and C).

Remarkably, the knockdown of endocan significantly attenuated the TGF-β-driven induction of all three lncRNAs. This evidence suggests that endocan expression is not only a downstream effect of TGF-β but also a crucial upstream regulator of the non-coding RNA circuitry in human cardiac fibroblasts. By modulating the levels of MALAT1, H19, and HOTAIR, endocan appears to integrate external growth factor signaling with internal epigenetic programs, thereby sustaining the transdifferentiation of fibroblasts into activated myofibroblasts (Supplementary Table S2).

## 3. Discussion

The evidence highlighting the role of endocan in renal fibrosis and the crucial function of TGF-β in driving the fibrotic response leads to the hypothesis that TGF-β signaling pathways could affect endocan expression throughout the fibrotic process. Herein, we studied the possible relationship between TGF-β signaling and endocan expression in human cardiac fibroblasts. We demonstrated that TGF-β is able to induce endocan expression and release in cardiac fibroblasts. Furthermore, by comparing results obtained from cardiac fibroblasts stimulated with TGF-β alone with those obtained from cardiac fibroblasts in which endocan knockdown was induced before TGF-β treatment, we found that endocan is able to modulate TGF-β-regulated pathways, thereby influencing the expression of important pro-fibrotic genes that are involved in ECM turnover and fibroblast activation. In addition, we also observed that endocan affects the expression of the lncRNAs H19, MALAT1, and HOTAIR, which are abundantly expressed in myocardial tissue and deregulated during fibrosis.

Specifically, our data suggest that endocan does not merely act as a downstream target of TGF-β but rather functions as a key component of a pro-fibrotic feed-forward loop that amplifies TGF-β signaling. To ground these findings in a clinical context, we first performed a secondary analysis of the GSE116250 transcriptomic dataset. Our results showed that endocan mRNA levels were significantly upregulated in the left ventricles of patients with DCM compared with non-failing donors. As shown above, endocan correlates with key markers of cardiac fibrosis, including TGFB1, ACTA2 (α-SMA), and COL1A1. These clinical findings suggest that endocan is a relevant player in the fibrotic remodeling of the human heart, providing a robust rationale for our in vitro investigations.

The ability of TGF-β to regulate endocan expression was previously reported in 2005 by Kirwan and colleagues [15]. In their study, they found that endocan was among the TGF-β responsive genes involved in ECM remodeling and cell proliferation in lamina cribrosa (LC) glial cells, a pro-fibrotic cell type regulating ECM turnover in LC, and playing a role in the development of primary open-angle glaucoma (POAG), a progressive optic neuropathy characterized by excessive ECM modification in the LC. Similarly, Hung et al., studying endocan in renal fibrosis, have found that ESM-1 overexpression promotes the expression of mesenchymal and fibrotic markers in mouse kidney MES13 cells. Additionally, they also discovered a time-dependent increase in endocan expression and these markers in mice kidney tissue samples, concluding that endocan promotes renal fibrosis by regulating the EMT pathways both in vitro and in vivo [29]. In a different fibrotic context, elevated serum endocan levels were also detected in patients with NAFLD and advanced liver fibrosis [27].

TGF-β is a key mediator of fibrosis, as it triggers the activation of fibroblasts into myofibroblasts, which are the major cells involved in the improper deposition of ECM molecules and secretion of MMPs, which in turn degrade the basement membrane, facilitating the entry of infiltrating cells [30,31]. In our study, using TGF-β as a pro-fibrotic stimulus, we demonstrated that endocan is part of TGF-β signaling in cardiac fibroblasts, and its expression correlates with the activation of pro-fibrotic signaling pathways, indicating a possible involvement of this PG in the mechanisms driving the development of cardiac fibrosis.

To support this hypothesis, we studied the degree of activation of intracellular mediators and TGF-β-regulated genes. Evaluating α-SMA and Col1a1, we found that in endocan knockdown cells, the effects of TGF-β were significantly reduced. A similar trend was observed when evaluating the degree of SMAD3 phosphorylation, a member of the SMAD proteins, which are the main signal transducers of TGF-β signaling [32].

In response to specific stimuli, including TGF-β, cardiac fibroblasts transdifferentiate into myofibroblasts, a more motile and contractile phenotype with an increased capacity to synthesize ECM proteins, including fibronectin and collagen [32,33,34]. These events shift the balance of ECM turnover and increase the synthesis and accumulation of fibrotic matrix, and the expression of smooth muscle cell markers such as α-SMA, absent in quiescent fibroblasts, is considered a hallmark of myofibroblast differentiation [32,35]. Based on these previous findings, our data suggest that endocan, by modulating SMAD3, may be involved in the myofibroblast transdifferentiation process promoted by TGF-β. It should be noted that, in our experimental setting, cell area was not systematically quantified, and no overt morphological change was apparent upon qualitative inspection of the immunofluorescence images following 24 h of TGF-β stimulation. This observation is consistent with previous studies showing that morphological remodeling associated with fibroblast-to-myofibroblast transdifferentiation, including cell flattening and stress fiber reorganization, becomes more evident at later time points (48–72 h), whereas molecular markers of myofibroblast activation are already detectable at 24 h [36]. Future studies including longer stimulation periods and quantitative morphometric analysis will be needed to fully characterize the morphological trajectory of endocan-dependent fibroblast activation.

Notably, TGF-β promotes cardiac fibrosis via the activation of the classical TGF-β/Smad signaling pathway and by regulating non-classical signaling pathways, including the PI3K/AKT and the ERK1/2 pathways [37]. In this setting, it has been reported that TGF-β, by activating AKT, promotes SMAD signaling and the transformation of cardiac fibroblasts into myofibroblasts; on the contrary, the attenuation of AKT signaling reduces the fibrotic response in mice [38]. Regarding the ERK1/2 pathway, it has been reported that its upregulation induced by TGF-β increases fibroblast proliferation and transdifferentiation and hence collagen production [39]. In our model, the degree of Akt and ERK 1/2 phosphorylation was significantly reduced in endocan knockdown cells. It should be noted that p-AKT and p-ERK1/2 levels were normalized to β-actin rather than to their respective total protein forms; therefore, we cannot formally exclude that part of the observed variation reflects changes in total kinase abundance. Consistently, immunofluorescence analysis revealed that endocan knockdown reduced SMAD3 activation and ERK1/2 phosphorylation in TGF-β-stimulated fibroblasts, independently corroborating the biochemical results obtained by Western blotting. Overall, in this experimental setting, these findings suggest that endocan orchestrates a complex signaling coordination, serving as a mandatory scaffold for the full activation of both Smad and non-Smad pathways. This finding aligns with emerging evidence that other proteoglycans can similarly act as required mediators of TGF-β receptor signaling; for instance, serglycin has been shown to be necessary for active TGF-β receptor I signaling and for the paracrine activation of stromal fibroblasts in a different pathological context [40]. Furthermore, these results are consistent with our previous study, in which we observed that endocan knockdown, by modulating the AKT and ERK 1/2 pathways affects cell proliferation and progression in NSCLC cells [17]. In this regard, it should be clarified that, in the present study, the term “coordination” refers to the parallel, endocan-dependent co-regulation of the canonical Smad and non-canonical AKT/ERK branches of TGF-β signaling, as demonstrated by the concurrent attenuation of SMAD3, AKT, and ERK1/2 phosphorylation following endocan knockdown. While these functional data support a model in which endocan acts as a shared molecular node required for the full activation of both signaling branches, they do not formally establish a direct reciprocal interaction between the Smad and AKT/ERK pathways themselves. Further mechanistic studies, including pathway-specific inhibition and epistasis experiments, will be required to dissect the precise molecular hierarchy linking endocan to these signaling branches. Cardiac fibroblasts, being the main source of structural ECM in cardiac tissue, produce several ECM regulatory proteins, such as MMPs and their inhibitors, orchestrating ECM turnover and homeostasis [41]. In activated cardiac fibroblasts and in myofibroblasts, MMPs expression and activity are increased, leading to excessive ECM degradation and promoting cardiac fibrosis. In our study we have evaluated MMP-3 and MMP-9 expression, selected as representative members of two distinct MMP subfamilies (stromelysins and gelatinases, respectively) with well-documented TGF-β-responsiveness and established roles in ECM degradation during cardiac remodeling [41,42]. We acknowledge that other MMPs may also contribute to endocan-dependent ECM remodeling in this setting. Notably, several MMPs, including MMP-2, MMP-7, MMP-8, MMP-12, MMP-13, and MT1-MMP, can themselves proteolytically activate latent TGF-β through cleavage of the latency-associated peptide (LAP) and latent TGF-β-binding proteins (LTBPs), establishing a potential feed-forward loop between MMP activity and TGF-β signaling [4]. Future studies investigating the role of endocan in regulating this broader repertoire of MMPs, as well as their endogenous inhibitors (TIMPs), would provide a more comprehensive picture of endocan-dependent ECM remodeling in cardiac fibrosis.

Comparing data obtained from cardiac fibroblasts stimulated with TGF-β alone with those obtained from endocan knockdown cardiac fibroblasts, we observed that MMP-3 and MMP-9 expression was significantly reduced in endocan knockdown cells stimulated with TGF-β, indicating that endocan is able to modulate the expression of these two ECM regulatory proteins in TGF-β-activated cardiac fibroblasts.

Increasing evidence underscores the role of lncRNAs in TGF-β-driven fibrosis [43]. Here we studied the effects of endocan knockdown on the expression of MALAT1, HOTAIR, and H19, three representative TGF-β-associated lncRNAs [43].

MALAT1 knockdown prevents fibroblast proliferation and ECM molecule production in angiotensin II-treated cardiac fibroblasts by regulating TGF-β signaling [44]. Additionally, in another study, it was reported that MALAT1 promotes lung fibrosis by targeting miR-503, leading to the activation of the PI3K/Akt pathway and promoting the EMT process [45]. Increased H19 expression contributes to cardiac fibroblast proliferation and fibrosis by regulating ERK1/2 activity [46]. Furthermore, the knockdown of H19 attenuates the expression of connective tissue growth factor (CTGF), thus leading to a decrease in Col1a1 and α-SMA in mouse primary cardiac fibroblasts [47]. The knockdown of HOTAIR repressed the ERK and JNK signaling pathways and consequently affected cell proliferation and migration and promoted fibrosis in atrial fibroblasts treated with angiotensin II [48]. Moreover, it has been proved that HOTAIR, via the TGF-β/Smad signaling pathway, could promote endometrial fibrosis [49].

Our findings revealed that the expression of these selected lncRNAs was increased after TGF-β treatment. Conversely, endocan knockdown significantly decreased the expression of MALAT1, H19 and HOTAIR compared with cells stimulated with TGF-β alone. This suggests that the profibrotic effects of endocan are also associated with the epigenetic reprogramming of the fibroblast, positioning endocan as a contributor to the lncRNA networks that sustain the chronic fibrotic response, likely via its upstream effects on TGF-β/Smad and AKT/ERK signaling rather than through a direct molecular interaction.

It should be noted that our data demonstrate a functional requirement for endocan in the TGF-β-driven induction of MALAT1, H19, and HOTAIR, but do not establish a direct molecular mechanism underlying this relationship. Given that endocan modulates the same upstream Smad and AKT/ERK pathways already implicated in the regulation of these lncRNAs, the observed effect is most likely indirect and pathway-dependent. Future studies employing RNA immunoprecipitation or luciferase reporter assays will be needed to determine whether endocan, or downstream effectors of its signaling, directly interacts with the regulatory regions or transcriptional machinery controlling these lncRNAs.

**Figure 9:**
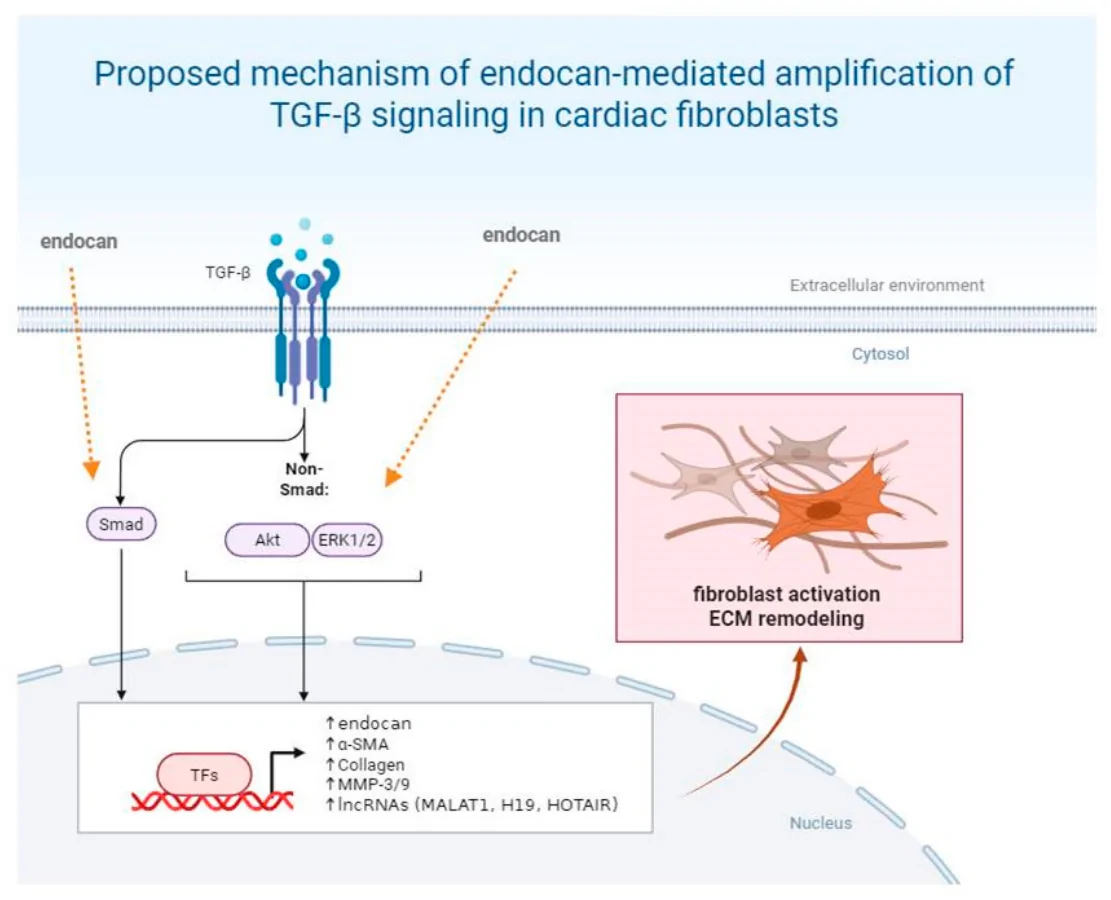
Proposed mechanism of endocan-mediated amplification of TGF-β signaling in cardiac fibroblasts. TGF-β triggers the activation of both canonical (SMAD3) and non-canonical (AKT and ERK) pathways. Endocan, induced by TGF-β, acts as a molecular facilitator in a positive feed-forward loop, sustaining the phosphorylation and activity of these signaling mediators. Orange dashed arrows indicate the facilitating action of endocan on Smad and AKT/ERK1/2 signaling; black solid arrows indicate downstream signal transduction toward the nucleus; the brown solid arrow indicates the resulting cellular outcome. This integrated response promotes the expression of pro-fibrotic markers (Col1a1, α-SMA) and extracellular matrix remodeling enzymes (MMP-3, MMP-9), while also modulating the expression of key lncRNAs (MALAT1, HOTAIR, H19). Collectively, these molecular events drive the transdifferentiation of cardiac fibroblasts into activated myofibroblasts, schematically represented as the orange-highlighted cell within the fibroblast activation panel. Created in BioRender. Scuruchi, M. (2026) https://BioRender.com/3u1agyr.

Taken together, our data suggest that endocan is involved in the TGF-β-regulated gene networks in cardiac fibroblasts. Moreover, endocan affects fibroblast behavior by regulating the expression of genes involved in ECM remodeling, fibroblast proliferation, and their transdifferentiation into myofibroblasts. This is in part due to its modulatory action on the TGF-β/Smad and TGF-β/AKT/ERK pathways, as well as on the expression of the lncRNAs MALAT1, H19 and HOTAIR, an effect that is most plausibly mediated indirectly through these same signaling pathways rather than via a direct molecular mechanism (Figure 9).

## 4. Materials and Methods

### 4.1. Materials

Recombinant human TGF-β1 was purchased from PeproTech (Rocky Hill, NJ, USA, Cat. #100-21). Endocan siRNA (ESM-1 siRNA), a non-targeting negative control siRNA (NC siRNA), and the RNAiMAX transfection kit were supplied by Life Technologies (Carlsbad, CA, USA). Fibroblast Basal Medium-2, Fibroblast Growth Supplement-2, fetal bovine serum (FBS), penicillin and streptomycin were purchased from Innoprot (Derio, Spain). Phosphate-buffered saline solution (PBS) and other general laboratory chemicals were obtained from Sigma-Aldrich S.r.l. (Milan, Italy).

### 4.2. Bioinformatic Analysis of Human Heart Dataset

To evaluate endocan expression and its relationship with fibrotic markers in the human heart, we performed a secondary analysis of a publicly available transcriptomic dataset (GSE116250) retrieved from the Gene Expression Omnibus (GEO) database. This dataset comprises RNA-sequencing data from left ventricle (LV) samples of 37 patients with Dilated Cardiomyopathy (DCM) and 14 non-failing donors (NF). Normalized gene expression values, expressed as RPKM (reads per kilobase of transcript per million mapped reads) as provided in the original dataset, were extracted for ESM1 (endocan), TGFB1, ACTA2 (α-SMA), and COL1A1. No additional transformation was applied prior to statistical analysis. Pearson’s correlation coefficients (r) and *p*-values were calculated. To account for potential confounding by clinical covariates, these correlation analyses were repeated using partial correlation adjusted for age and sex, following the covariate-adjustment approach described in the original dataset publication [50]. Briefly, endocan and each fibrotic marker were independently regressed on age and sex, and the residuals of these regressions were used to compute the adjusted Pearson correlation coefficient. A sensitivity analysis was additionally performed using Cook’s distance (threshold = 4/n) to identify and exclude potentially influential observations, and correlations were recalculated on the reduced dataset.

### 4.3. Cell Cultures

Human cardiac fibroblasts (P10452-HCF) from adult human hearts were purchased from Innoprot (Derio, Spain). Cells were grown in a humidified incubator with 5% CO_2_ at 37 °C in Fibroblast Basal Medium-2 supplemented with 5% FBS, a solution of streptomycin and penicillin, and Fibroblast Growth Supplement-2. The culture medium was replaced every 2–3 days. Experiments were carried out with fibroblast cultures between the third and tenth passages.

### 4.4. Cell Treatments

After seeding in six-well culture plates (1.5 × 105 cells/well), the culture medium was replaced with OPTIMEM (Life Technologies, Carlsbad, CA, USA) after 24 h. Cells were then transfected with the endocan siRNA using the RNAiMAX transfection kit (Life Technologies, Carlsbad, CA, USA) according to the manufacturer’s instructions. A non-targeting negative control siRNA (NC siRNA) was also used under the same conditions. After 24 h of incubation, cells were treated with TGF-β (10 ng/mL). Finally, cells and medium were collected for biochemical evaluations 24 h after the last treatment.

### 4.5. RNA Isolation, cDNA Synthesis, and qPCR Analysis

Total RNA was extracted from cardiac fibroblasts using TRIzol reagent (Thermo Fisher Scientific, Waltham, MA, USA), according to the manufacturer’s instructions. Reverse transcription was performed using the High-Capacity cDNA Reverse Transcription Kit (Thermo Fisher Scientific, Waltham, MA, USA), following the manufacturer’s protocol. Quantitative real-time PCR (qPCR) was carried out using an Applied Biosystems 7500 Real-Time PCR System (Applied Biosystems, Foster City, CA, USA) with PowerUp SYBR Green Master Mix (Thermo Fisher Scientific, Waltham, MA, USA) with the specific primer pairs listed in Table 1. GAPDH was used as the endogenous control for normalization in all qPCR experiments. Relative gene expression was calculated using the 2^−ΔΔCt^ method.

### 4.6. Western Blot

Proteins were extracted from cells using RIPA buffer (Cell Signaling, Danvers, MA, USA), supplemented with a protease and phosphatase inhibitor cocktail (Sigma-Aldrich, Milano, Italy). After protein determination using the Bio-Rad protein assay system (Bio-Rad, Hercules, CA, USA), aliquots of 20 μg of protein were separated by SDS-PAGE and transferred onto PVDF membranes (Amersham Bioscience, Buckinghamshire, UK). Then, the membranes were washed with TBS containing 0.1% Tween 20 buffer, blocked with a blotting buffer containing 2% BSA, and incubated overnight at 4 °C with the specific primary antibodies. Next, the membranes were incubated with the secondary polyclonal antibodies (7076, 1:3000, Cell Signaling, Danvers, MA, USA) (7074, 1:500; Cell Signaling, Danvers, MA, USA) in TBS containing 0.15% Tween 20 buffer for 1 h. The membranes were then washed with a wash buffer solution, and images were acquired and quantified using a bio-image analysis system (C-DiGit, Licor, Lincoln, NE, USA). Primary antibodies used included endocan (H00011082-M02, 1:1000; Abnova, Taipei, Taiwan), phospho-Akt (13038, 1:1000; Cell Signaling, Danvers, MA, USA), phospho-ERK 1/2 (9101, 1:1000; Cell Signaling, Danvers, MA, USA), Col1a1 (72026, 1:1000; Cell Signaling, Danvers, MA, USA), α-SMA (ab32575, 1:1000; Abcam, Cambridge, UK), phospho-SMAD3 (ab52903,1:1000; Abcam, Cambridge, UK), and β-actin (ab8226, 1:1000; Abcam, Cambridge, UK), the latter used as an endogenous control. Densitometric values for each target protein were normalized to β-actin, and the resulting ratio was further normalized to the corresponding CTRL sample from the same experiment (set as 1), consistent with recommended practices for Western blot densitometric quantification [51].

### 4.7. Immunofluorescence

The cells, seeded onto the glass coverslips Nunc™ Lab-Tek™ Chamber Slides (Thermo Fisher Scientific, Waltham, MA, USA), were fixed in 4% paraformaldehyde in 0.2 M phosphate buffer (pH 7.4) for 30 min at room temperature (RT), and then they were washed three times for 10 min each with phosphate-buffered saline (PBS). To block nonspecific binding sites and to permeabilize the membranes, the cells were preincubated with 0.3% Triton X-100 in PBS for 10 min and with 1% bovine serum albumin (BSA) in PBS for 20 min at RT. Then, cells were incubated with a rabbit anti-phospho-SMAD3 monoclonal antibody (ab52903,1:200; Abcam, Cambridge, UK), or a rabbit anti-phospho-ERK 1/2 (9101, 1:500; Cell Signaling, Danvers, MA, USA) polyclonal antibody, overnight at 4 °C. After numerous rinses, the primary antibody was detected using a FITC-conjugated IgG anti-rabbit (Jackosn ImmunoReseach Laboratories, Inc., West Grove, PA, USA) for 1 h at RT. Nuclei were stained with DAPI diluted 1:1000 in PBS for 10 min at RT. Finally, cells were washed in PBS, and the coverslips were mounted on slides. Immunofluorescence reactions were analyzed, and images were acquired using a Zeiss LSM 5 Duo (Carl Zeiss, Jena, Germany) confocal laser scanning microscope. All images were digitalized at a resolution of eight bits into an array of 2048 × 2048 pixels. Optical sections of fluorescence specimens were obtained using a HeNe laser (λ 543 nm) at a 762 s scanning speed with up to eight averages. Contrast and brightness were established by examining the brightest-labeled pixels and choosing the settings that allowed clear visualization of the structural details while keeping the pixel intensity at its highest (200). Each image was acquired within 62 s to minimize photodegradation, and five microscopic fields for each magnification were taken. Digital images were cropped, and figure montages were prepared using Adobe Photoshop 7.0 (Adobe System, Palo Alto, CA, USA). Immunofluorescence images were used exclusively for qualitative assessment of the protein staining pattern and subcellular distribution. No quantitative analysis of nuclear fluorescence intensity or nuclear-to-cytoplasmic ratio was performed, and no z-stack acquisition or three-dimensional reconstruction was carried out.

### 4.8. Endocan, MMP-3 and MMP-9 ELISA Assay

At 24 h after TGF-β treatment, the culture supernatants were collected, particulate matter was removed by centrifugation, and a protease inhibitor cocktail and 1 nM PMSF were added. Endocan levels were assayed using a commercial ELISA kit provided by RayBiotech (#ELH-ESM1, Peachtree Corners, GA, USA), while MMP-3 and MMP-9 levels were detected by commercial ELISA kits provided by R&D Systems (#DMP300, #DMP900, Minneapolis, MN, USA), in accordance with the manufacturer’s instructions. The absorbance of each sample was measured through a photometer for microplate reading (DAS, Palombara Sabina, Italy) at λ 450 nm. Endocan and MMP-9 protein levels are reported in pg/mL, while MMP-3 protein levels are reported in ng/mL.

### 4.9. Statistical Analysis

Data are reported as the mean ± SD from at least five independent experiments for qPCR and ELISA assays and three independent experiments for Western blot analysis. Statistical analysis was conducted using a one-way analysis of variance (ANOVA) followed by Tukey’s post hoc test. A *p*-value of < 0.05 was considered statistically significant. Student’s *t*-test and Pearson’s correlation were used for the secondary bioinformatic analysis. GraphPad Prism software (version 7.04 for Windows; GraphPad Software, San Diego, CA, USA) was used to generate the graphs.

## 5. Conclusions

These insights highlight the regulatory role of endocan in the metabolic shift that cardiac fibroblasts undergo in response to fibrotic stimuli. Since endocan is a secreted molecule, our findings indicate that it is functionally required for this process and also suggest its potential as a circulating biomarker for monitoring fibrotic progression. Targeting endocan could represent a promising therapeutic strategy for mitigating fibrosis. However, further studies are needed to better investigate the biochemical mechanisms in order to fully confirm this hypothesis.

## Figures and Tables

**Table 1:** qPCR primers.

Gene	Forward Primer (5′→3′)	Reverse Primer (5′→3′)
*ESM-1*	CTGATCCCGGCTGTGATTT	TCTGGATCCACCATGCATC
*MMP-3*	ATCTCTTCCTTCAGGCGTGG	ATCCAGCTCGTACCTCATTTCC
*MMP-9*	CTTCCAGTACCGAGAGAAAGC	ATGTCATAGGTCACGTAGCCCA
*HOTAIR*	GCACTCACAGACAGAGGTTTA	CTCTGTACTCCCGTTCCCTAGA
*H19*	CCAGAACCCACAACATGAAAG	TCACCTTCCAGAGCCGATT
*MALAT1*	GGAAAGCGAGTGGTTGGTA	ATCCCTTTACACCTCAGTACGA
*GAPDH*	CACCAGGGCTGCTTTTAACTCT	ATCTCGCTCCTGGAAGATGGT

## Data Availability

The data presented in this study are available on request from the corresponding authors.

## Supplementary Materials

The following supporting information can be downloaded at: https://www.mdpi.com/article/10.3390/ijms27177745/s1.

## Abbreviations

The following abbreviations are used in this manuscript:

TGF-β	Transforming Growth Factor-β
lncRNAs	Long noncoding RNAs
EMT	Epithelial–mesenchymal transition
MMP-3	Matrix metalloproteinase-3
MMP-9	Matrix metalloproteinase-9
Col1a1	Collagen type I alpha 1
α-SMA	α-Smooth muscle actin
ESM-1	Endothelial cell-specific molecule 1
HOTAIR	HOX transcript antisense RNA
H19	Imprinted maternally expressed transcript
MALAT-1	Metastasis-associated lung adenocarcinoma transcript 1.
PG	Proteoglycan
IM-HCF	Immortalized Human Cardiac Fibroblasts
VEGF-A	Vascular Endothelial Growth Factor A
VEGF-C	Vascular Endothelial Growth Factor C
EGFR	Epidermal Growth Factor Receptor
TNF-α	Tumor Necrosis Factor alpha
IL-1β	Interleukin 1 beta
NF-kB	Nuclear Factor kappa-light-chain-enhancer of activated B cells
PI3K/Akt	Phosphoinositide 3-kinase/protein kinase B
PKC	Protein Kinase C
ERK1/2	Extracellular signal-Regulated Kinases 1/2
NAFLD	Nonalcoholic Fatty Liver Disease
mRNA	Messenger RNA
ELISA	Enzyme-Linked Immunosorbent Assay
siRNA	small interfering RNA
SMAD3	SMAD Family Member 3
AKT	Protein kinase B
qPCR	Quantitative Polymerase Chain Reaction
DSPG	Dermatan Sulfate Proteoglycan
DS	Dermatan Sulfate
Endocan	Endothelial-specific molecules-1
LC	Lamina Cribrosa
POAG	Primary Open-Angle Glaucoma
MMPs	Matrix Metalloproteinases
NSCLC	Non-Small Cell Lung Cancer
CTGF	Connective Tissue Growth Factor
JNK	c-Jun N-terminal Kinases

## References

[1] Deng Z., Fan T., Xiao C., Tian H., Zheng Y., Li C., He J. (2024). TGF-β signaling in health, disease and therapeutics. Signal Transduct. Target. Ther..

[2] Su J., Morgani S.M., David C.J., Wang Q., Er E.E., Huang Y.H., Basnet H., Zou Y., Shu W., Soni R.K. (2020). TGF-β orchestrates fibrogenic and developmental EMTs via the RAS effector RREB1. Nature.

[3] Chakravarthy A., Khan L., Bensler N.P., Bose P., De Carvalho D.D. (2018). TGF-β-associated extracellular matrix genes link cancer-associated fibroblasts to immune evasion and immunotherapy failure. Nat. Commun..

[4] Piperigkou Z., Mangani S., Kremmydas S., Koletsis N.E., Karamanos N.K. (2026). A guide to the types, structures, and multifaceted functions of matrix metalloproteinases in cancer. FEBS J..

[5] Dobaczewski M., Bujak M., Li N., Gonzalez-Quesada C., Mendoza L.H., Wang X.F., Frangogiannis N.G. (2010). Smad3 signaling critically regulates fibroblast phenotype and function in healing myocardial infarction. Circ. Res..

[6] Lassalle P., Molet S., Janin A., Heyden J.V., Tavernier J., Fiers W., Devos R., Tonnel A.B. (1996). ESM-1 is a novel human endothelial cell-specific molecule expressed in lung and regulated by cytokines. J. Biol. Chem..

[7] Wellner M., Herse F., Janke J., Gorzelniak K., Engeli S., Bechart D., Lasalle P., Luft F.C., Sharma A.M. (2003). Endothelial cell specific molecule-1—A newly identified protein in adipocytes. Horm. Metab. Res..

[8] Seftor E.A., Meltzer P.S., Schatteman G.C., Gruman L.M., Hess A.R., Kirschmann D.A., Seftor R.E., Hendrix M.J. (2002). Expression of multiple molecular phenotypes by aggressive melanoma tumor cells: Role in vasculogenic mimicry. Crit. Rev. Oncol..

[9] Scuruchi M., Aliquò F., Avenoso A., Mandraffino G., Vermiglio G., Minuti A., Campo S., Campo G.M., D’Ascola A. (2023). Endocan Knockdown Down-Regulates the Expression of Angiogenesis-Associated Genes in Il-1ß Activated Chondrocytes. Biomolecules.

[10] Kali A., Shetty K.S. (2014). Endocan: A novel circulating proteoglycan. Indian J. Pharmacol..

[11] Karamanos N.K., Piperigkou Z., Theocharis A.D., Watanabe H., Franchi M., Baud S., Brézillon S., Götte M., Passi A., Vigetti D. (2018). Proteoglycan Chemical Diversity Drives Multifunctional Cell Regulation and Therapeutics. Chem. Rev..

[12] Piperigkou Z., Mangani S., Koletsis N.E., Koutsakis C., Mastronikolis N.S., Franchi M., Karamanos N.K. (2026). Principal mechanisms of extracellular matrix-mediated cell-cell communication in physiological and tumor microenvironments. FEBS J..

[13] Chen J., Jiang L., Yu X.H., Hu M., Zhang Y.K., Liu X., He P., Ouyang X. (2021). Endocan: A Key Player of Cardiovascular Disease. Front. Cardiovasc. Med..

[14] Delehedde M., Devenyns L., Maurage C.A., Vives R.R. (2013). Endocan in cancers: A lesson from a circulating dermatan sulfate proteoglycan. Int. J. Cell Biol..

[15] Kirwan R.P., Leonard M.O., Murphy M., Clark A.F., O’Brien C.J. (2005). Transforming growth factor-beta-regulated gene transcription and protein expression in human GFAP-negative lamina cribrosa cells. Glia.

[16] Li L., Zhao Q., Kong W. (2018). Extracellular matrix remodeling and cardiac fibrosis. Matrix Biol..

[17] Aliquò F., Minuti A., Avenoso A., Mandraffino G., Campo G.M., Campo S., D’Ascola A., Scuruchi M. (2023). Endocan Promotes Pro-Tumorigenic Signaling in Lung Cancer Cells: Modulation of Cell Proliferation, Migration and lncRNAs H19 and HULC Expression. Int. J. Mol. Sci..

[18] Abid M.R., Yi X., Yano K., Shih S.C., Aird W.C. (2006). Vascular endocan is preferentially expressed in tumor endothelium. Microvasc. Res..

[19] Scuruchi M., D’Ascola A., Avenoso A., Mandraffino G., Campo S., Campo G.M. (2021). Endocan, a novel inflammatory marker, is upregulated in human chondrocytes stimulated with IL-1 beta. Mol. Cell. Biochem..

[20] Wang Y., Sun X. (2020). The functions of LncRNA in the heart. Diabetes Res. Clin. Pract..

[21] Bhan A., Soleimani M., Mandal S.S. (2017). Long Noncoding RNA and Cancer: A New Paradigm. Cancer Res..

[22] Lai Y., He S., Ma L., Lin H., Ren B., Ma J., Zhu X., Zhuang S. (2017). HOTAIR functions as a competing endogenous RNA to regulate PTEN expression by inhibiting miR-19 in cardiac hypertrophy. Mol. Cell. Biochem..

[23] Liu L., An X., Li Z., Song Y., Li L., Zuo S., Liu N., Yang G., Wang H., Cheng X. (2016). The H19 long noncoding RNA is a novel negative regulator of cardiomyocyte hypertrophy. Cardiovasc. Res..

[24] Puthanveetil P., Gutschner T., Lorenzen J. (2020). MALAT1: A therapeutic candidate for a broad spectrum of vascular and cardiorenal complications. Hypertens. Res..

[25] Viereck J., Bührke A., Foinquinos A., Chatterjee S., Kleeberger J.A., Xiao K., Janssen-Peters H., Batkai S., Ramanujam D., Kraft T. (2020). Targeting muscle-enriched long non-coding RNA H19 reverses pathological cardiac hypertrophy. Eur. Heart J..

[26] Nie X., Fan J., Wang D.W. (2023). The Function and Therapeutic Potential of lncRNAs in Cardiac Fibrosis. Biology.

[27] Klisić A., Kavarić N., Abenavoli L., Stanišić V., Spasojević-Kalimanovska V., Kotur-Stevuljević J., Ninić A. (2020). Is endocan a novel potential biomarker of liver steatosis and fibrosis?. J. Med. Biochem..

[28] Tok D., Ekiz F., Basar O., Coban S., Ozturk G. (2014). Serum endocan levels in patients with chronic liver disease. Int. J. Clin. Exp. Med..

[29] Hung T.W., Chu C.Y., Yu C.L., Lee C.C., Hsu L.S., Chen Y.S., Hsieh Y.H., Tsai J.P. (2020). Endothelial Cell-Specific Molecule 1 Promotes Endothelial to Mesenchymal Transition in Renal Fibrosis. Toxins.

[30] Ku J.C., Raiten J., Li Y. (2024). Understanding fibrosis: Mechanisms, clinical implications, current therapies, and prospects for future interventions. Biomed. Eng. Adv..

[31] Parks W.C., Wilson C.L., López-Boado Y.S. (2004). Matrix metalloproteinases as modulators of inflammation and innate immunity. Nat. Rev. Immunol..

[32] Fan D., Takawale A., Lee J., Kassiri Z. (2012). Cardiac fibroblasts, fibrosis and extracellular matrix remodeling in heart disease. Fibrogenesis Tissue Repair.

[33] Jiang N., Zhang Z., Shao X., Jing R., Wang C., Fang W., Mou S., Ni Z. (2020). Blockade of thrombospondin-1 ameliorates high glucose-induced peritoneal fibrosis through downregulation of TGF-β1/Smad3 signaling pathway. J. Cell. Physiol..

[34] Li X., Zhang Z.L., Wang H.F. (2017). Fusaric acid (FA) protects heart failure induced by isoproterenol (ISP) in mice through fibrosis prevention via TGF-β1/SMADs and PI3K/AKT signaling pathways. Biomed. Pharmacother..

[35] Shinde A.V., Humeres C., Frangogiannis N.G. (2017). The role of α-smooth muscle actin in fibroblast-mediated matrix contraction and remodeling. Biochim. Biophys. Acta Mol. Basis Dis..

[36] Marconi G.D., Fonticoli L., Rajan T.S., Lanuti P., Della Rocca Y., Pierdomenico S.D., Trubiani O., Pizzicannella J., Diomede F. (2021). Transforming Growth Factor-Beta1 and Human Gingival Fibroblast-to-Myofibroblast Differentiation: Molecular and Morphological Modifications. Front. Physiol..

[37] Ma Z.G., Yuan Y.P., Wu H.M., Zhang X., Tang Q.Z. (2018). Cardiac fibrosis: New insights into the pathogenesis. Int. J. Biol. Sci..

[38] Ma Z.G., Yuan Y.P., Zhang X., Xu S.C., Wang S.S., Tang Q.Z. (2017). Piperine Attenuates Pathological Cardiac Fibrosis via PPAR-γ/AKT Pathways. EBioMedicine.

[39] Peng H., Carretero O.A., Peterson E.L., Rhaleb N.E. (2010). Ac-SDKP inhibits transforming growth factor-beta1-induced differentiation of human cardiac fibroblasts into myofibroblasts. Am. J. Physiol. Heart Circ. Physiol..

[40] Manou D., Golfinopoulou M.A., Alharbi S.N.D., Alghamdi H.A., Alzahrani F.M., Theocharis A.D. (2024). The Expression of Serglycin Is Required for Active Transforming Growth Factor β Receptor I Tumorigenic Signaling in Glioblastoma Cells and Paracrine Activation of Stromal Fibroblasts via CXCR-2. Biomolecules.

[41] Wang Y., Jiao L., Qiang C., Chen C., Shen Z., Ding F., Lv L., Zhu T., Lu Y., Cui X. (2024). The role of matrix metalloproteinase 9 in fibrosis diseases and its molecular mechanisms. Biomed. Pharmacother..

[42] Scuruchi M., Mannino F., Imbesi C., Pallio G., Vermiglio G., Bagnato G., Minutoli L., Bitto A., Squadrito F., Irrera N. (2023). Biglycan Involvement in Heart Fibrosis: Modulation of Adenosine 2A Receptor Improves Damage in Immortalized Cardiac Fibroblasts. Int. J. Mol. Sci..

[43] Tang P.C., Zhang Y.Y., Li J.S., Chan M.K., Chen J., Tang Y., Zhou Y., Zhang D., Leung K.T., To K.F. (2022). LncRNA-Dependent Mechanisms of Transforming Growth Factor-β: From Tissue Fibrosis to Cancer Progression. Non-Coding RNA.

[44] Huang S., Zhang L., Song J., Wang Z., Huang X., Guo Z., Chen F., Zhao X. (2019). Long noncoding RNA MALAT1 mediates cardiac fibrosis in experimental postinfarct myocardium mice model. J. Cell. Physiol..

[45] Yan W., Wu Q., Yao W., Li Y., Liu Y., Yuan J., Han R., Yang J., Ji X., Ni C. (2017). MiR-503 modulates epithelial-mesenchymal transition in silica-induced pulmonary fibrosis by targeting PI3K p85 and is sponged by lncRNA MALAT1. Sci. Rep..

[46] Tao H., Cao W., Yang J.J., Shi K.H., Zhou X., Liu L.P., Li J. (2016). Long noncoding RNA H19 controls DUSP5/ERK1/2 axis in cardiac fibroblast proliferation and fibrosis. Cardiovasc. Pathol..

[47] Huang Z.W., Tian L.H., Yang B., Guo R.M. (2017). Long Noncoding RNA H19 Acts as a Competing Endogenous RNA to Mediate CTGF Expression by Sponging miR-455 in Cardiac Fibrosis. DNA Cell Biol..

[48] Tan W., Wang K., Yang X., Wang K., Wang N., Jiang T.-B. (2022). LncRNA HOTAIR promotes myocardial fibrosis in atrial fibrillation through binding with PTBP1 to increase the stability of Wnt5a. Int. J. Cardiol..

[49] Wu J., Jin L., Zhang Y., Duan A., Liu J., Jiang Z., Huang L., Chen J., Liu Z., Lu D. (2020). LncRNA HOTAIR promotes endometrial fibrosis by activating TGF-β1/Smad pathway. Acta Biochim. Biophys. Sin..

[50] Sweet M.E., Cocciolo A., Slavov D., Jones K.L., Sweet J.R., Graw S.L., Reece T.B., Ambardekar A.V., Bristow M.R., Mestroni L. (2018). Transcriptome analysis of human heart failure reveals dysregulated cell adhesion in dilated cardiomyopathy and activated immune pathways in ischemic heart failure. BMC Genom..

[51] Gassmann M., Grenacher B., Rohde B., Vogel J. (2009). Quantifying Western blots: Pitfalls of densitometry. Electrophoresis.

