# Supplementary material for: Endocan Participates in TGF-β/Smad and AKT/ERK Signaling Coordination and lncRNA Modulation in Human Cardiac Fibroblasts
Source: Int J Mol Sci. 2026 Aug 29;27(17):7745. doi: 10.3390/ijms27177745 (PMC13566329; doi:10.3390/ijms27177745)
Supplement: Supplementary file 1 [file ijms-27-07745-s001.zip › Supplementari Figure S1_WB Biological Replicates.pdf]

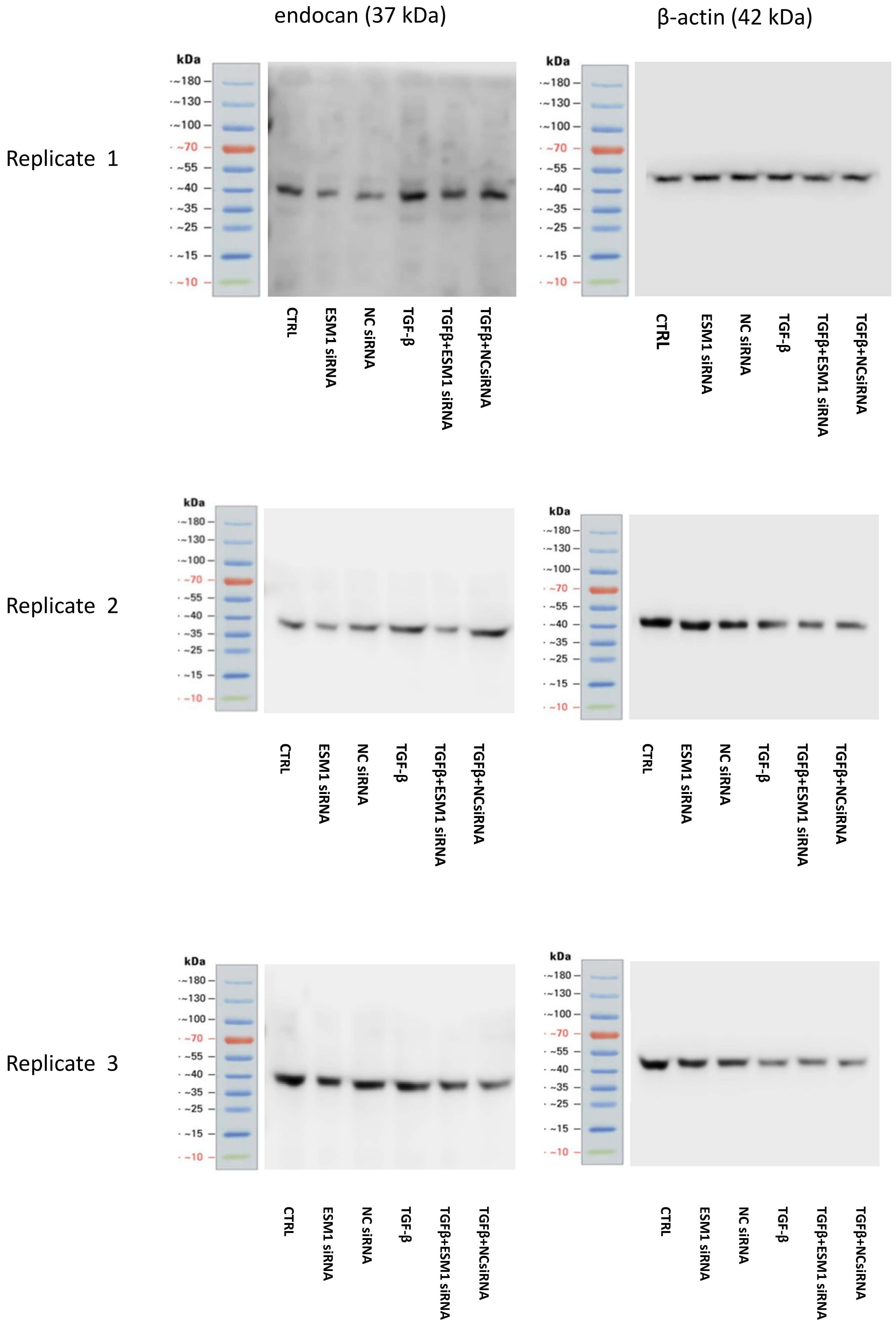

endocan (37 kDa)
β-actin (42 kDa)
Replicate 1
Replicate 2
Replicate 3
kDa
~180
~130
~100
~70
~55
~40
~35
~25
~15
~10
CTRL
ESM1 siRNA
NC siRNA
TGF-β
TGFβ+ESM1 siRNA
TGFβ+NCsiRNA

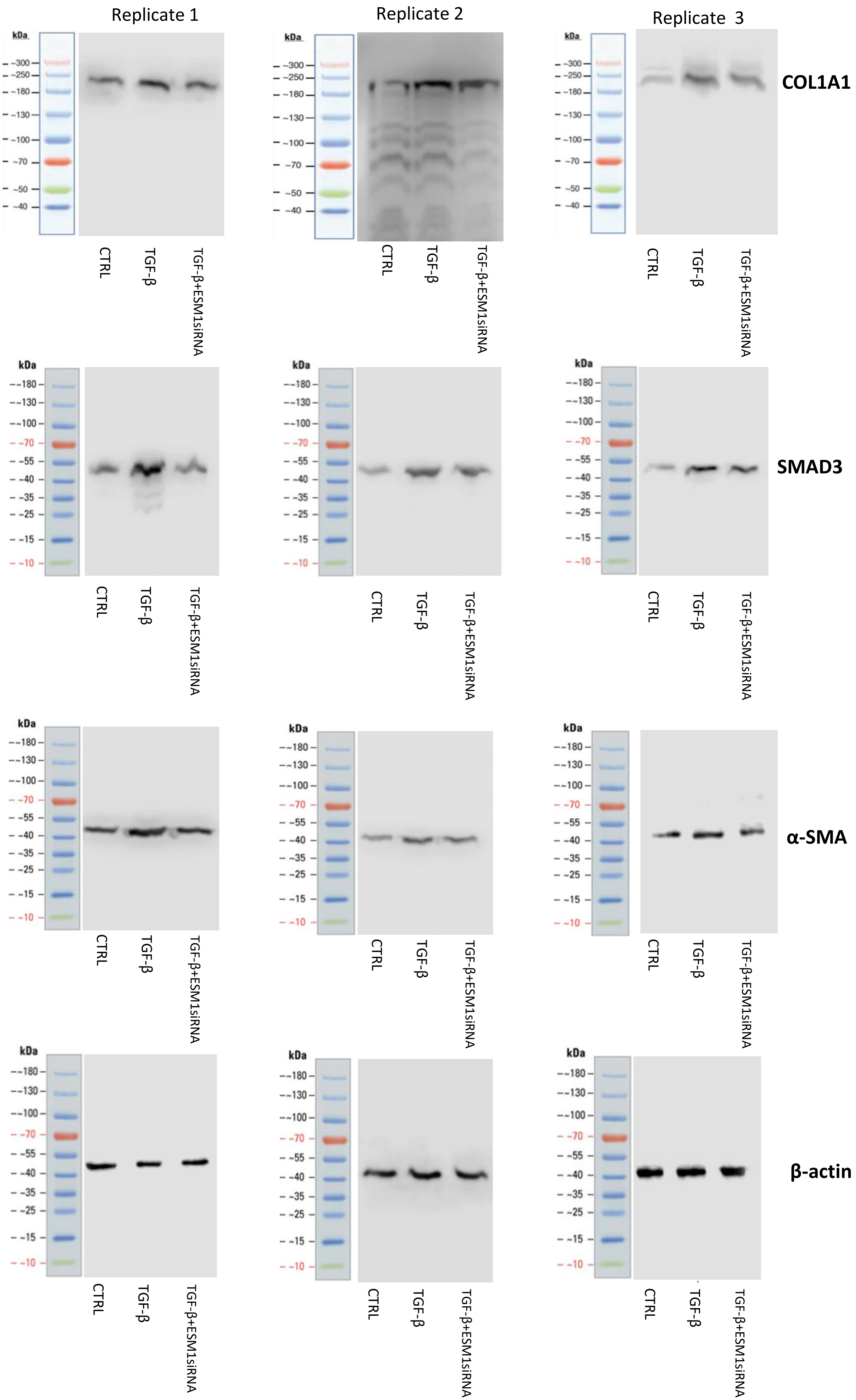
Replicate 1
Replicate 2
Replicate 3
COL1A1
SMAD3
α-SMA
β-actin
kDa
~300
~250
~180
~130
~100
~70
~55
~50
~40
~35
~25
~15
~10
CTRL
TGF-β
TGF-β+ESM1siRNA

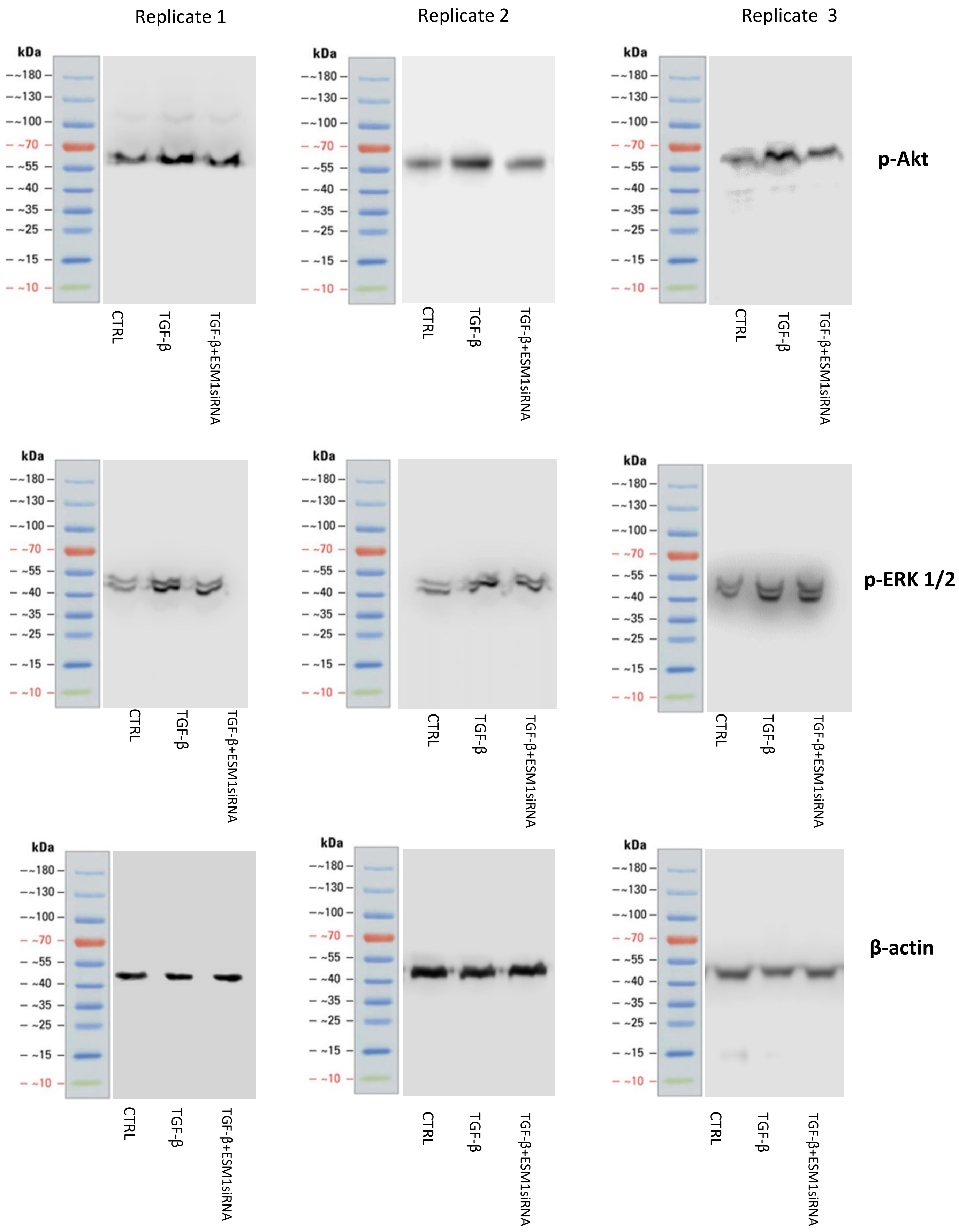

Replicate 1
Replicate 2
Replicate 3
p-Akt
p-ERK 1/2
β-actin
kDa
~180
~130
~100
~70
~55
~40
~35
~25
~15
~10
CTRL
TGF-β
TGF-β+ESM1siRNA
