# Supplementary material for: Endocan Participates in TGF-β/Smad and AKT/ERK Signaling Coordination and lncRNA Modulation in Human Cardiac Fibroblasts
Source: Int J Mol Sci. 2026 Aug 29;27(17):7745. doi: 10.3390/ijms27177745 (PMC13566329; doi:10.3390/ijms27177745)
Supplement: Supplementary file 1 [file ijms-27-07745-s001.zip › Supplementary_Table_S1.pdf]

Supplementary Table S1. Sensitivity and covariate-adjustment analysis of transcriptomic correlations with endocan.

Marker	Raw r	Raw p	Adjusted r (age+sex)	Adjusted p	Excluded samples (Cook's D)	r after exclusion	p after exclusion
**TGFB1**	0.594	<0.0001	0.597	<0.0001	DCM19, DCM75	0.482	0.0005
**ACTA2**	0.583	<0.0001	0.583	<0.0001	DCM19, DCM75	0.410	0.0035
**COL1A1**	0.653	<0.0001	0.653	<0.0001	NF10, DCM8, DCM19, DCM75	0.817	<0.0001

Pearson correlation coefficients between endocan (ESM1) expression and TGFB1, ACTA2, and COL1A1 expression in the GSE116250 dataset (n = 51; 14 non-failing donors and 37 patients with dilated cardiomyopathy), before ("Raw") and after ("Adjusted") partial correlation adjustment for age and sex. Sensitivity analysis was performed using Cook's distance (threshold = 4/n) to identify influential observations; correlation coefficients after exclusion of these observations are reported in the last two columns.
