# Supplementary material for: Endocan Participates in TGF-β/Smad and AKT/ERK Signaling Coordination and lncRNA Modulation in Human Cardiac Fibroblasts
Source: Int J Mol Sci. 2026 Aug 29;27(17):7745. doi: 10.3390/ijms27177745 (PMC13566329; doi:10.3390/ijms27177745)
Supplement: Supplementary file 1 [file ijms-27-07745-s001.zip › Supplementary_Table_S2.pdf]

Supplementary Table S2. Individual raw data underlying qPCR, ELISA and Western blot quantifications.

Individual biological replicate values used to generate the bar graphs shown in the corresponding main-text figures. All values are rounded to two decimal places.

A. qPCR raw data (fold-change relative to CTRL)

Figure 2A. Endocan (ESM1) mRNA (qPCR)

Rep	CTRL	ESM1 siRNA	NC siRNA	TGF-β	TGF-β+ESM1 siRNA	TGF-β+NC siRNA
1	1.00	0.50	1.10	3.20	2.20	3.20
2	1.00	0.25	0.90	2.82	1.60	3.85
3	1.00	0.40	1.15	3.90	1.20	3.97
4	1.00	0.30	0.75	3.30	1.80	2.70
5	1.00	0.15	0.85	3.40	1.70	2.80
6	1.00	0.45	0.65	3.70	1.90	3.00

Figure 7A. MMP-3 mRNA (qPCR)

Rep	CTRL	TGF-β	TGF-β+ESM1 siRNA
1	1.00	2.74	2.77
2	1.00	3.72	2.20
3	1.00	3.22	1.50
4	1.00	2.90	1.78
5	1.00	3.93	1.53
6	1.00	3.29	2.21

Figure 7C. MMP-9 mRNA (qPCR)

Rep	CTRL	TGF-β	TGF-β+ESM1 siRNA
1	1.00	4.43	1.77
2	1.00	5.32	3.38
3	1.00	3.19	2.30
4	1.00	4.26	2.30
5	1.00	3.15	2.65
6	1.00	2.87	1.95

Figure 8A. MALAT1 (qPCR)

Rep	CTRL	TGF-β	TGF-β+ESM1 siRNA
1	1.00	7.26	2.85
2	1.00	6.26	1.85
3	1.00	7.50	3.09
4	1.00	8.88	4.48
5	1.00	6.10	1.73

Figure 8B. H19 (qPCR)

Rep	CTRL	TGF-β	TGF-β+ESM1 siRNA
1	1.00	33.09	12.92
2	1.00	35.63	11.56
3	1.00	25.80	13.71
4	1.00	27.40	12.25
5	1.00	33.88	14.40
6	1.00	37.00	15.04
7	1.00	38.83	8.56

Figure 8C. HOTAIR (qPCR)

Rep	CTRL	TGF-β	TGF-β+ESM1 siRNA
1	1.00	14.84	6.90
2	1.00	11.48	8.41
3	1.00	15.03	7.30
4	1.00	10.21	4.90
5	1.00	18.21	6.27
6	1.00	12.22	5.70

B. ELISA raw data

Figure 2C. Endocan secreted (pg/mL)

Rep	CTRL	siRNA	NCsiRNA	TGF-β	TGF-β+siRNA	TGF-β+NCsiRNA
1	34.00	24.00	35.00	90.00	35.00	92.00
2	44.00	15.00	52.00	70.00	45.00	60.00
3	32.00	14.00	26.00	85.00	50.00	90.00
4	31.00	17.00	32.00	74.00	38.00	68.00
5	34.00	12.00	40.00	88.00	58.00	91.00

Figure 7B. MMP-3 secreted (ng/mL)

Rep	CTRL	TGF-β	TGF-β+siRNA
1	50.00	100.00	60.00
2	40.00	80.00	72.00
3	55.00	90.00	80.00
4	40.00	120.00	45.00
5	55.00	92.00	50.00

Figure 7D. MMP-9 secreted (pg/mL)

Rep	CTRL	TGF-β	TGF-β+siRNA
1	80.00	160.00	100.00
2	75.00	150.00	135.00
3	78.00	156.00	98.00
4	86.00	172.00	120.00
5	60.00	120.00	105.00

C. Western blot densitometry — raw band intensities and normalized values

Raw values represent Integrated Density (target and β-actin) measured for each replicate. Normalized values = (Target/β-actin ratio) relative to the CTRL of the same replicate (CTRL = 1). Raw density values are rounded to the nearest integer; normalized values are rounded to two decimal places.

Figure 2B. Endocan protein

Group	Rep	β-actin (raw)	Target (raw)	Normalized (CTRL=1)
CTRL	1	14500	14800	1.00
CTRL	2	11200	8600	1.00
CTRL	3	15000	11000	1.00
siRNA	1	16200	7630	0.46
siRNA	2	15000	4780	0.42
siRNA	3	14500	7700	0.72
NCsiRNA	1	15000	9730	0.64
NCsiRNA	2	15300	8050	0.69
NCsiRNA	3	13800	10200	1.01
TGF	1	16600	24300	1.43
TGF	2	11000	12500	1.48
TGF	3	9190	11500	1.71
TGF+siRNA	1	14500	15600	1.05
TGF+siRNA	2	7300	6060	1.08
TGF+siRNA	3	9430	8700	1.26
TGF+NCsiRNA	1	13000	20200	1.52
TGF+NCsiRNA	2	9600	12800	1.74
TGF+NCsiRNA	3	7900	8200	1.42

Figure 3B. COL1A1 protein

Group	Rep	β-actin (raw)	Target (raw)	Normalized (CTRL=1)
CTRL	1	1350	19000	1.00
CTRL	2	15690	17500	1.00
CTRL	3	1770	38000	1.00
TGF	1	864	28000	2.30
TGF	2	17716	56000	2.83
TGF	3	1850	81000	2.04
TGF+siRNA	1	983	17000	1.23
TGF+siRNA	2	15910	30500	1.72
TGF+siRNA	3	1870	60000	1.49

Figure 3C. SMAD3 protein

Group	Rep	β-actin (raw)	Target (raw)	Normalized (CTRL=1)
CTRL	1	1350	12500	1.00
CTRL	2	15690	12800	1.00
CTRL	3	1770	3200	1.00
TGF	1	864	29900	3.74
TGF	2	17716	58600	4.05
TGF	3	1850	10200	3.05

TGF+siRNA	1	983	26500	2.91
TGF+siRNA	2	15910	24800	1.91
TGF+siRNA	3	1870	7650	2.26

Figure 3A. α-SMA protein

Group	Rep	β-actin (raw)	Target (raw)	Normalized (CTRL=1)
CTRL	1	1350	113000	1.00
CTRL	2	15690	59000	1.00
CTRL	3	1770	1010	1.00
TGF	1	864	203000	2.81
TGF	2	17716	125500	1.88
TGF	3	1850	2100	1.99
TGF+siRNA	1	983	134000	1.63
TGF+siRNA	2	15910	86600	1.45
TGF+siRNA	3	1870	1300	1.22

Figure 5A. p-Akt protein

Group	Rep	β-actin (raw)	Target (raw)	Normalized (CTRL=1)
CTRL	1	13150	74700	1.00
CTRL	2	67700	3650	1.00
CTRL	3	44100	4430	1.00
TGF	1	11400	150000	2.32
TGF	2	60000	7120	2.20
TGF	3	37500	10000	2.65
TGF+siRNA	1	13160	110000	1.47
TGF+siRNA	2	67400	4500	1.24
TGF+siRNA	3	44200	7460	1.68

Figure 5B. p-ERK1/2 protein

Group	Rep	β-actin (raw)	Target (raw)	Normalized (CTRL=1)
CTRL	1	13150	97200	1.00
CTRL	2	67700	90700	1.00
CTRL	3	44100	132000	1.00
TGF	1	11400	157700	1.87
TGF	2	60000	142000	1.77
TGF	3	37500	260000	2.32
TGF+siRNA	1	13160	117000	1.20
TGF+siRNA	2	67400	129000	1.43
TGF+siRNA	3	44200	142200	1.07
